# Transport of Eicosapentaenoic Acid-Derived PGE_3_, PGF_3α_, and TXB_3_ by ABCC4

**DOI:** 10.1371/journal.pone.0109270

**Published:** 2014-10-02

**Authors:** Nobuaki Tanaka, Hiroaki Yamaguchi, Nariyasu Mano

**Affiliations:** Department of Pharmaceutical Sciences, Tohoku University Hospital, Sendai, Japan; Technion-Israel Institute of Technology, Israel

## Abstract

**Background:**

Eicosapentaenoic acid-derived prostaglandin (PG) E_3_, PGF_3α_, and thromboxane (TX) B_3_ are bioactive lipid mediators which have anti-cancer and anti-inflammatory effects. To exert their effects, PGE_3_, PGF_3α_, and TXB_3_ must be released to the extracellular space from cells, but the release mechanism has been unclear. We therefore investigated the contribution of ATP-binding cassette transporter C4 (ABCC4), which has been known as a prostanoids efflux transporter, to the release of PGE_3_, PGF_3α_, and TXB_3_.

**Materials and Methods:**

ATP-dependent transport of PGE_3_, PGF_3α_, and TXB_3_ via ABCC4 was investigated by using inside-out membrane vesicles prepared from ABCC4-overexpressing HEK293 cells. To evaluate the contribution of ABCC4 to the release of PGE_3_, PGF_3α_, and TXB_3_, we measured the extracellular and intracellular levels of PGE_3_, PGF_3α_, and TXB_3_ in A549 cells when we used ABCC4 inhibitors (dipyridamole, MK571, and probenecid) or ABCC4 siRNAs. The quantification of PGE_3_, PGF_3α_, and TXB_3_ was performed by using liquid chromatography-tandem mass spectrometry.

**Results:**

The apparent *K_m_* values for ABCC4-mediated transport were 2.9±0.1 µM for PGE_3_, 12.1±1.3 µM for PGF_3α_, and 11.9±1.4 µM for TXB_3_ and the ATP-dependent accumulation of PGE_3_, PGF_3α_, and TXB_3_ into vesicles was decreased by using typical substrates and inhibitors of ABCC4. ABCC4 inhibitors and ABCC4 knockdown showed the reduction of extracellular/intracellular ratio of PGE_3_ (40–60% of control) and PGF_3α_ (60–80% of control) in A549 cells.

**Conclusions:**

Our results suggest that PGE_3_, PGF_3α_, and TXB_3_ are substrates of ABCC4 and ABCC4 partially contributes to the release of PGE_3_ and PGF_3α_.

## Introduction

Eicosapentaenoic acid (EPA), one of omega-3 polyunsaturated fatty acids, has beneficial effects on various diseases, such as cardiovascular diseases and cancer [1]–[4]. EPA is incorporated into membrane phospholipids in an esterified form and, after extracellular stimuli, cytosolic phospholipase A_2_ (cPLA_2_) releases EPA as a free acid form into the intracellular space [5]. EPA not only competes with arachidonic acid (AA) in metabolic pathways but is also converted into several bioactive lipid mediators [5]–[7].

3-Series prostanoids, including prostaglandin (PG) E_3_, PGD_3_, PGF_3α_, PGI_3_, and thromboxane (TX) A_3_, are bioactive EPA metabolites synthesized via the cyclooxygenase (COX) pathway [8]. The beneficial effects of the 3-series prostanoids have been gradually clarified, especially PGE_3_ showed anti-inflammatory and anti-cancer effects through competition with PGE_2_ [9]–[11]. It was reported that the *K_m_* and EC_50_ values of 3-series prostanoids for their receptors were higher than those of AA-derived 2-series prostanoids, supporting that 3-series prostanoids could work as partial agonists [11], [12]. Before binding to their cell surface receptors, 3-series prostanoids need to be released to the extracellular space from cells. Therefore, the release process of 3-series prostanoids may be associated with the regulation of signaling by the 3-series prostanoids, but the release mechanism of 3-series prostanoids has not been focused on.

ATP-binding cassette transporter C4 (ABCC4) is a member of the multidrug resistance-associated protein (MRP) family and transports a variety of endogenous compounds such as cyclic nucleotides, bile acids, and eicosanoids [13]–[15], suggesting that ABCC4 may be associated with the maintenance of homeostasis [16]. Previous reports using ABCC4-overexpressing inside-out membrane vesicles showed that several prostanoids (PGE_1_, PGE_2_, PGF_2α_, and TXB_2_) were transported by ABCC4 with high affinity [17], [18]. Further studies conducted in many laboratories have revealed the importance of ABCC4 on the release of PGE_2_, PGF_2α_, and TXB_2_ [19], [20], thus ABCC4 is known as a prostanoids efflux transporter. Since 3-series prostanoids have chemical structures similar to the 2-series prostanoids, 3-series prostanoids might also be transported by ABCC4.

The purpose of our study is to investigate whether 3-series prostanoids (PGE_3_, PGF_3α_, and TXB_3_) are released from intracellular to extracellular space by ABCC4. To achieve this purpose, we first determined that 3-series prostanoids were substrates of ABCC4 by using inside-out membrane vesicles. Furthermore, the contribution of ABCC4 to the release of 3-series prostanoids was investigated by analyses of the extracellular and intracellular levels of 3-series prostanoids of human lung epithelial A549 cells.

## Materials and Methods

### Chemicals

All prostanoids (PGE_3_, PGF_3α_, TXB_3_, PGE_2_, PGF_2α_, and TXB_2_), deuterated prostanoids (PGE_2_-d4, PGF_2α_-d4, and TXB_2_-d4), and EPA were purchased from Cayman Chemical Co. (Ann Arbor, MI). Calcium ionophore A23187 and indomethacin were purchased from Sigma Aldrich (St. Louis, MO). Adenosine 5′-triphosphate (ATP) disodium salt hydrate, 5′-adenylic acid (AMP), and sodium creatine phosphate hydrate were purchased from Tokyo Chemical Industry Co., Ltd. (Tokyo, Japan). Creatine kinase was purchased from Roche Applied Science (Tokyo, Japan). Dipyridamole, probenecid, quercetin, methotrexate, and folic acid were purchased from Wako Pure Chemical Industries (Osaka, Japan). MK571, celecoxib, candesartan, estradiol 17β-glucronide (E_2_17βG), adenosine 3′, 5′-cyclic monophosphate (cAMP), and guanosine 3′, 5′-cyclic monophosphate (cGMP) were purchased from Sigma Aldrich (St. Louis, MO). All other chemicals were of the highest purity available.

### Cell culture

Human lung adenocarcinoma epithelial A549 cells were obtained from American Type Culture Collection (Rockville, MD). ABCC4-overexpressing human embryonic kidney HEK293 cells (HEK293/4.63) and control HEK293 cells (HEK293/P.B.) were kindly provided by Dr. Piet Borst (Netherlands Cancer Institute). The cells were grown in Dulbecco's modified Eagle's medium (DMEM) with 10% fetal bovine serum (ICN Biomedicals, Inc, Aurora, OH) at 37°C under 5% CO_2_.

### Sample preparation

A549 cells were cultured on 60 mm dish (4×10^5^ cells/dish) for 72 h to confluent. After confluency should be confluence, A549 cells were incubated for 24 h in serum-free DMEM containing EPA (100 µM). After 24 h, A549 cells were exposed to calcium ionophore A23187 (10 µM) and each of ABCC4 inhibitors (50 µM dipyridamole, 50 µM MK571, or 500 µM probenecid) in serum-free DMEM for 5 min. The medium was collected as extracellular samples. The cells were scraped into ice-cold PBS (containing 10 µM indomethacin) and centrifuged at 1,500×*g* for 5 min at 4°C, the resulting pellet was collected as intracellular samples. All samples were stored at −80°C until analysis. Both extracellular and intracellular samples were purified by using Bond Elut C18 solid-phase extraction cartridges (Agilent Technologies, Santa Clara, CA) as described previously [21].

### Preparation of inside-out membrane vesicles

The preparation of ABCC4-expressing inside-out membrane vesicles from HEK293/4.63cells was carried out by previous method [20]. HEK293/4.63 cells were collected and incubated in hypotonic buffer (0.5 mM sodium phosphate, 0.1 mM EDTA, 2 mM PMSF, pH 7.4) at 4°C for 90 min. The suspension was centrifuged at 100,000×*g* for 40 min at 4°C, and the pellet was suspended in ice-cold TS buffer (50 mM Tris-HCl, 250 mM sucrose, pH 7.4) and homogenized 25 times with a tight fitting Dounce homogenizer. The homogenate was centrifuged at 500×*g* for 10 min at 4°C. The supernatant was centrifuged at 100,000×*g* for 40 min at 4°C, the resulting pellet was resuspended in TS buffer. The suspension was passed 25 times through a 27-gauge needle. The vesicles were dispensed in aliquots, frozen in liquid nitrogen, and stored at −80°C until assay.

### Vesicular transport assay

The vesicular transport assays were carried out by the rapid filtration method [20]. Membrane vesicles (25 µg) were incubated with reaction mixture (4 mM ATP or AMP, 10 mM MgCl_2_, 10 mM creatine phosphate, 100 µg/mL creatine kinase in TS buffer) at 37°C. The final volume was 50 µL. The transport was terminated by dilution into 1 mL of ice-cold TS buffer and immediately filtrated through a cellulose acetate membrane filter (OE67, 0.45 µm, 25 mm; GE Healthcare). The filter was washed with 3 mL ice-cold TS buffer. Prostanoids retained on the filter were harvested with 1 mL methanol containing 1 ng/mL of each internal standard (PGE_2_-d4, PGF_2α_-d4, and TXB_2_-d4). The methanol solutions were stored at −80°C until analysis. The time-dependent transport assays were conducted at the time of 0, 0.5, 1, 2, 5 min at the concentration of 1 µM (PGE_3_) or 2.5 µM (PGF_3α_ and TXB_3_). The concentration-dependent transport assays were conducted by exposure to 0.5, 1, 2.5, 5, 10, 20 µM (PGE_3_) or 2.5, 5, 10, 20, 40, 60 µM (PGF_3α_ and TXB_3_) for 0.5 min incubation. The inhibition studies were conducted at the concentration of 1 µM (PGE_3_) or 2.5 µM (PGF_3α_ and TXB_3_) in the presence or absence of each substrate or inhibitor. All substrates and inhibitors were dissolved in DMSO and the final concentration of DMSO was less than 1%. The kinetic parameters *K_m_* (Michaelis constant) and *V_max_* (maximum uptake velocity) were calculated by fitting the data of the prostanoids uptake rate to Michaelis-Menten equation.

### Quantification of prostanoids

The samples in methanol described above were dried under a nitrogen gas stream and the residue was reconstituted in 50 µL of mobile phase. The quantification of prostanoids was performed by using LC/MS/MS system described previously [21].

### ABCC4 small interfering RNA (siRNA) and siRNA transfection

Transfection of siRNAs (1 nM each) was performed by reverse transfection method using Lipofectamine RNAiMAX (Invitrogen). ABCC4 siRNAs (HSS115675 and HSS173510) or negative control (Stealth RNAi Negative Control Low GC Duplex) were obtained from Invitrogen. Each siRNA diluted in Opti-MEM I Reduced Serum medium and Lipofectamine RNAiMAX were mixed gently and incubated at room temperature for 15 min. These mixtures were added to each 60 mm dish and A549 cells suspended in antibiotics-free DMEM (4×10^5^ cells/dish) were added. The cells were incubated for 72 h at 37°C under 5% CO_2_ and then used for experiments.

### Western blotting

The cells were harvested with ice-cold PBS and centrifuged at 1,500×*g*, for 5 min at 4°C. The resulting pellet was suspended in ice-cold lysis buffer (1% Triton X-100, 0.1% sodium dodecyl sulfate (SDS), and 4.5 M urea). The suspension was allowed to stand for 5 min on ice and sonicated for 15 min at 4°C, and then centrifuged at 14,000×*g* for 15 min at 4°C.The supernatant was collected and a Bio-Rad Protein Assay was used to quantify the protein concentration of the collected supernatant. Proteins (5 µg/lane) were subjected to SDS-PAGE and transferred onto nitrocellulose membranes. The membranes were incubated with blocking buffer (0.05% Tween 20, 5% skim milk in PBS) and then incubated overnight with each primary antibodies in diluent buffer (0.05% Tween 20, and 0.05% skim milk in PBS) at room temperature. The primary antibodies were as follows: rat anti-ABCC4 monoclonal antibody (Clone M4I-10, Abcam), and mouse anti-actin monoclonal antibody (Clone C4/MAB1501, Chemicon, Temecula, CA). The proteins bound to antibodies were detected by using horseradish peroxidase-conjugated secondary antibodies (Santa Cruz Biotechnology, Santa Cruz, CA) and visualized by enhanced chemiluminescence (Amersham Biosciences Corp., Piscataway, NJ).

### Statistical analyses

Data are presented as means with S.E. Statistical significance among means of more than two groups was evaluated using ANOVA followed by Dunnett's test. Statistical significance was defined as *p*<0.05.

## Results

### ATP-dependent transport of PGE_3_, PGF_3α_, and TXB_3_ by ABCC4

**Figure 1:**
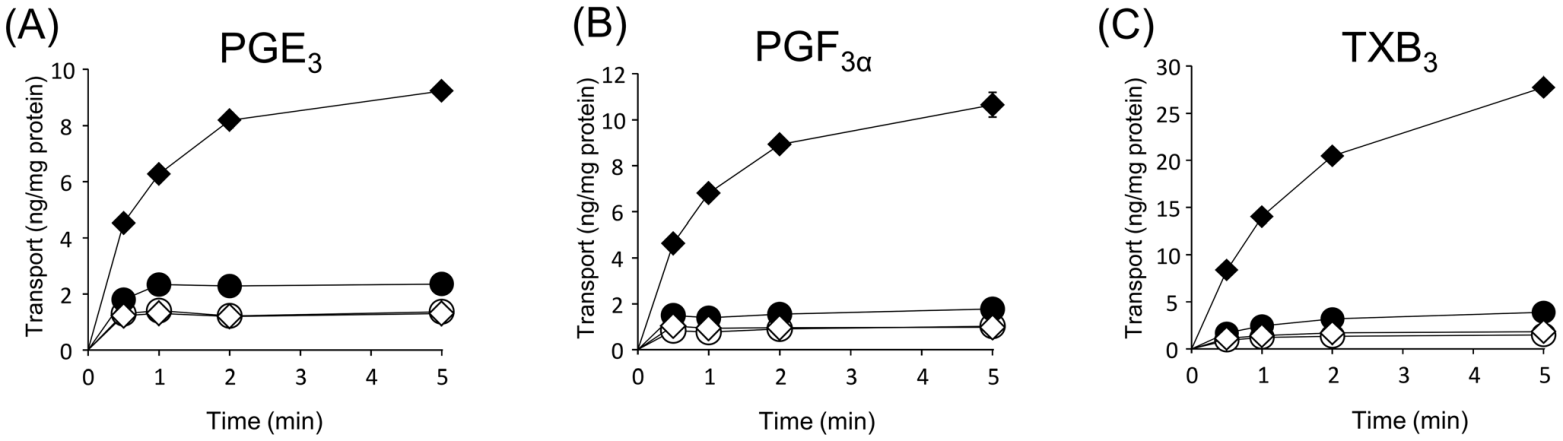
Time-dependent uptake of PGE_3_, PGF_3α_, and TXB_3_ transport by ABCC4. Membrane vesicles (25 µg) from HEK293/P.B. cells (circles) or HEK293/4.63 cells (diamonds) were incubated at 37°C with (A) 1 µM PGE_3_, (B) 2.5 µM PGF_3α_, and (C) 2.5 µM TXB_3_ in the presence of ATP (filled symbols) or AMP (open symbols). Each point represents the mean ± S.E. (n = 3).

We first examined whether 3-series prostanoids (PGE_3_, PGF_3α_, and TXB_3_) were transported by ABCC4 by using inside-out membrane vesicles prepared from HEK293/4.63 cells (ABCC4-overexpressing) and HEK293/P.B. cells (control). As shown in Figure 1, the ATP-dependent transport of 3-series prostanoids was rapid, and almost reached a steady state at 5 min. The amount of the ATP-dependent accumulation of PGE_3_, PGF_3α_, and TXB_3_ was 8.0-, 12.9-, 10.8-fold higher, respectively in vesicles prepared from HEK293/4.63 cells than in vesicles prepared from HEK293/P.B. cells at the time of 5 min. Following vesicular transport experiments were carried out at the time of 0.5 min.

### Kinetics of PGE_3_, PGF_3α_, and TXB_3_ transport by ABCC4

**Figure 2:**
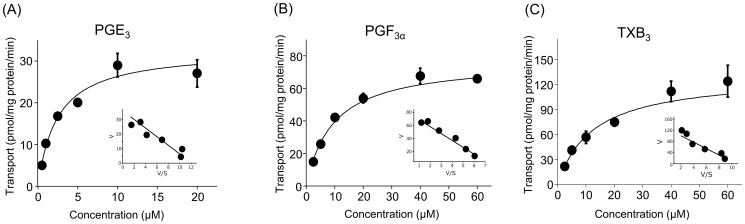
Concentration-dependent uptake of PGE_3_, PGF_3α_, and TXB_3_ transport by ABCC4. Membrane vesicles (25 µg) from HEK293/P.B. cells or HEK293/4.63 cells were incubated at 37°C at the time of 0.5 min in the presence of (A) PGE_3_, (B) PGF_3α_, and (C) TXB_3_. Rates of ATP-dependent transport were calculated by subtracting the uptake in the presence of AMP from that in the presence of ATP for three independent experiments. Each point represents the mean ± S.E. (n = 3). Representative experiments are shown. Inset: Eadie Hofstee plot.

To characterize the ABCC4-mediated transport activity of 3-series prostanoids, we examined the initial rate of their concentration-dependent transport during a 0.5 min incubation. ATP-dependent uptake approximated Michaelis Menten kinetics and the *K_m_* values of PGE_3_, PGF_3α_, and TXB_3_ were 2.9±0.1, 12.1±1.3, and 11.9±1.4 µM, and the corresponding *V_max_* values were 30.7±7.8, 65.9±13.2, and 117.2±11.2 pmol/mg protein/min, respectively (Figure 2, Table 1). The *K_m_* values of the 2-series prostanoids obtained by the same procedure were lower than those of the 3-series prostanoids (0.9±0.3 µM for PGE_2_, 5.3±0.7 µM for PGF_2α_, and 8.0±1.6 µM for TXB_2_, Table 1).

**Table 1:** Kinetic parameters of ABCC4-mediated prostanoids.

	*K_m_* (µM)	*V_max_* (pmol/mg protein/min)	*V_max_*/*K_m_* (µL/mg protein/min)	Reference
PGE_3_	2.9±0.1	30.7±7.8	10.6	Present study
PGF_3α_	12.1±1.3	65.9±13.2	5.4	Present study
TXB_3_	11.9±1.4	117.2±11.2	9.8	Present study
PGE_2_	0.9±0.3	7.1±1.3	7.9	Present study
	3.4	6.4	-	[17]
	3.5±0.2	3.25±0.2	0.9	[18]
PGF_2α_	5.3±0.7	52.5±3.2	9.9	Present study
	12.6±0.4	46.1±9.5	3.6	[18]
TXB_2_	8.0±1.6	51.6±3.1	6.5	Present study
	9.9±0.2	51±4.7	5.1	[18]

Rates of ATP-dependent transport were calculated by subtracting the uptake in the presence of AMP from that in the presence of ATP for three independent experiments. The values of *K_m_* and *V_max_* were calculated from Eadie-Hofstee plots. Data represent mean ± S.E. (n = 3).

### Inhibition of ABCC4-mediated PGE_3_, PGF_3α_, and TXB_3_ transport

We next carried out inhibition experiments of the ATP-dependent transport of the 3-series prostanoids by using typical substrates and inhibitors of ABCC4. The concentration of substrates and inhibitors was determined by the *K_m_* or IC_50_ values in a previous report [16]. As shown in Table 2, all of the substrates and inhibitors similarly reduced the transport of each 3-series prostanoids into vesicles. The reduction of the 3-series prostanoids transport by probenecid was small, which was similar to previous results [20]. Although cAMP and cGMP were transported by ABCC4 with the *K_m_* values of 45 µM and 10 µM, respectively in a previous study [13], 1000 µM cAMP and cGMP resulted in a reduction of transport by only 10–30% and 25–50%, respectively. The reason might be due to lower clearance of these cyclic nucleotides than that of 3-series prostanoids (0.09–0.21 versus 5.4–10.6 µL/mg protein/min) [13].

**Table 2:** Inhibition of ABCC4-mediated 3-series prostanoids transport.

Inhibitors	Concentration (µM)	ATP-dependent transport (% of control)		
		PGE_3_ (1 µM)	PGF_3α_ (2.5 µM)	TXB_3_ (2.5 µM)
Dipyridamole	10	59±8**	49±4**	51±1**
	50	20±4**	26±6**	23±1**
MK571	10	24±1**	30±3**	21±2**
	50	6±1**	6±4**	3±1**
Probenecid	100	102±0	104±2	92±2
	500	86±1	74±4*	68±4**
Quercetin	5	29±3**	37±2**	25±1**
Indomethacin	10	28±4**	42±8**	25±2**
Celecoxib	100	74±1*	69±7*	72±2**
Candesartan	50	64±6**	64±3*	56±2**
Methotrexate	200	42±1**	53±3**	30±3**
Folic acid	200	46±2**	37±4**	49±2**
E_2_17βG	50	30±5**	34±1**	30±3**
cAMP	1000	82±3	89±9	69±3**
cGMP	1000	64±5**	75±1	50±3**
PGE_2_	10	10±4**	22±4**	24±9**
PGF_2α_	10	36±5**	44±3**	41±3**
TXB_2_	10	61±1**	57±5**	61±1**

Membrane vesicles (25 µg) from HEK293 or HEK293/4.63 cells were incubated with PGE_3_ (1 µM), PGF_3α_ (2.5 µM), or TXB_3_ (2.5 µM) at 37°C. Rates of ATP-dependent transport were calculated by subtracting the uptake in the presence of AMP from that in the presence of ATP. Transport is expressed as percent of uptake in the absence of inhibitor. Each value represents the mean ± S.E. (n = 3).

*; *p*<0.05.

**; *p*<0.01.

### Inhibition of PGE_3_, PGF_3α_, and TXB_3_ release from A549 cells

**Figure 3:**
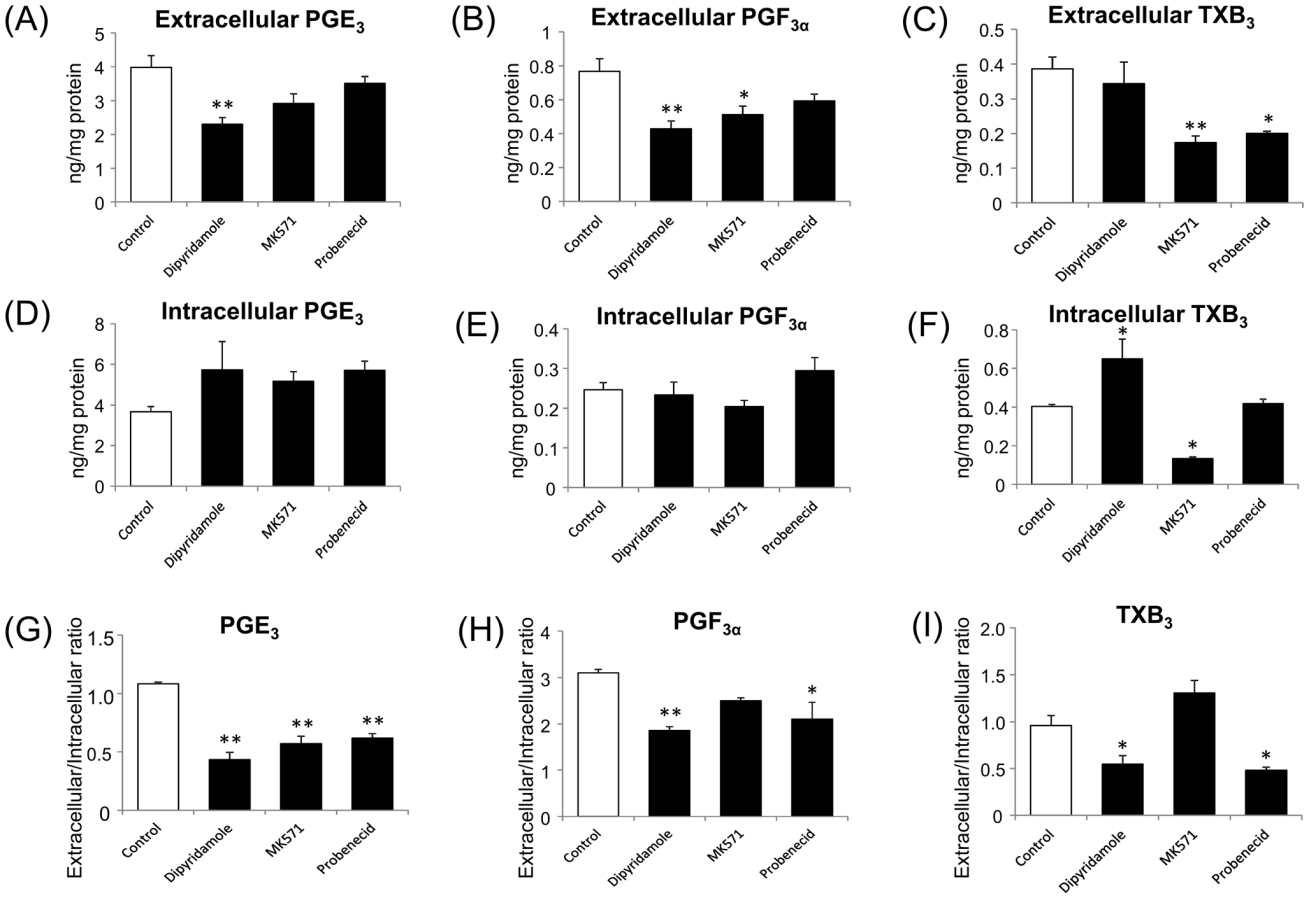
Effects of ABCC4 inhibitors on the transport of 3-series prostanoids in A549 cells. A549 cells were treated with 100 µM EPA for 24 h then 10 µM A23187 in presence or absence of ABCC4 inhibitors (50 µM dipyridamole, 50 µM MK571, or 500 µM probenecid) for 5 min. The extracellular and intracellular levels of (A, D) PGE_3_, (B, E) PGF_3α_, and (C, F) TXB_3_ were measured and (G–H) the ratio of extracellular to intracellular levels were calculated. Each column represents the mean with S.E. (n = 3). Representative experiments are shown. *; *p*<0.05, **; *p*<0.01.

To elucidate the contribution of ABCC4 to the release of 3-series prostanoids from cells, we carried out inhibition experiments by using A549 cells, which are reported to produce 3-series prostanoids and express ABCC4 [20], [21]. A549 cells were exposed with 100 µM EPA for 24 h before experiments [22]. Actually, the plasma concentration of EPA in healthy subjects ranges from 0.4 to 270 µM [23]–[26], so 100 µM EPA is clinically possible. A549 cells were treated with the calcium ionophore A23187 to stimulate prostanoid production. On the basis of the results of our vesicular transport study, 50 µM dipyridamole (as a mild inhibitor), 50 µM MK571 (as a strong inhibitor), and 500 µM probenecid (as a weak inhibitor) were used in the experiments. The extracellular levels of PGE_3_ and PGF_3α_ underwent the strongest decrease by dipyridamole (approximately 60% of control), followed by MK571 (approximately 70% of control), probenecid (approximately 80–90% of control) (Figure 3(A)–(B)). On the other hand, dipyridamole did not produce significant decrease of the extracellular levels of TXB_3_ (89% of control). A significant reduction of the extracellular levels of TXB_3_ was only observed when we used MK571 and probenecid (45% and 52% of control, respectively) (Figure 3(C)).

Although the intracellular levels of PGE_3_ showed a tendency to increase (155%, 141%, and 155% of control when dipyridamole, MK571, and probenecid were used, respectively), those of PGF_3α_ did not (94%, 82%, and 119% of control when dipyridamole, MK571, and probenecid were used, respectively) (Figure 3(D)–(E)). The intracellular levels of TXB_3_ were significantly increased by dipyridamole (160% of control) but were significantly decreased by MK571 (33% control), and probenecid did not change the intracellular levels of TXB_3_ (103% of control) (Figure 3(F)).

To eliminate the possibility of a decline in prostanoid production, the extracellular/intracellular ratio was calculated [19]. These inhibitors showed a larger reduction of the extracellular/intracellular ratio of PGE_3_ (40–60% of control) than those of PGF_3α_ (60–80% of control) (Figure 3(G)–(H)). On the other hand, the extracellular/intracellular ratio of TXB_3_ was decreased by dipyridamole and probenecid (57% and 50% of control, respectively), but not by MK571 (136% of control) (Figure 3(I)).

### Effects of ABCC4 knockdown on the release of PGE_3_, PGF_3α_, and TXB_3_

**Figure 4:**
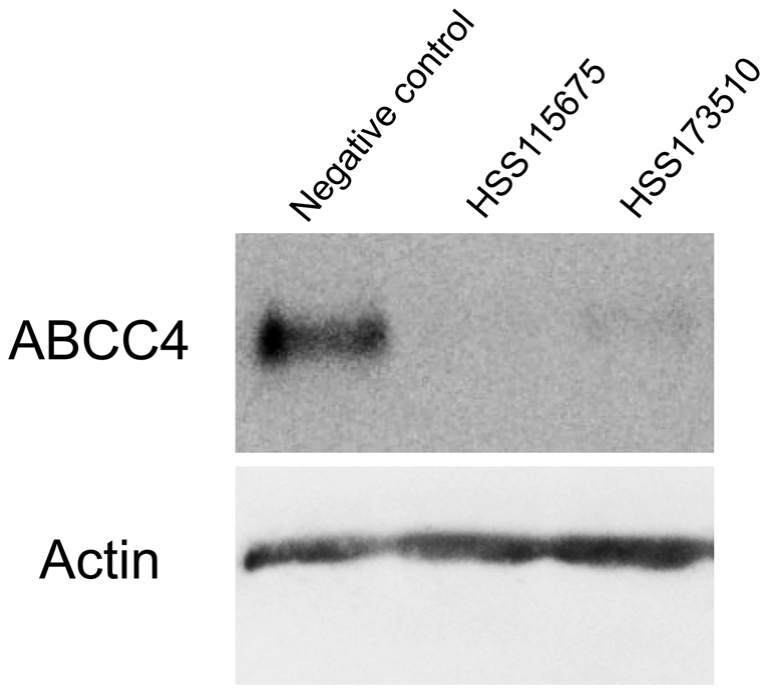
Representative Western blots for ABCC4 and actin. ABCC4 siRNAs (HSS115675 and HSS173510) and negative control siRNA were transfected in A549 cells for 72 h. Protein (5 µg/lane) was subjected to SDS-PAGE and then transferred onto nitrocellulose membranes. ABCC4 and actin were detected with monoclonal ABCC4 or actin antibody.

**Figure 5:**
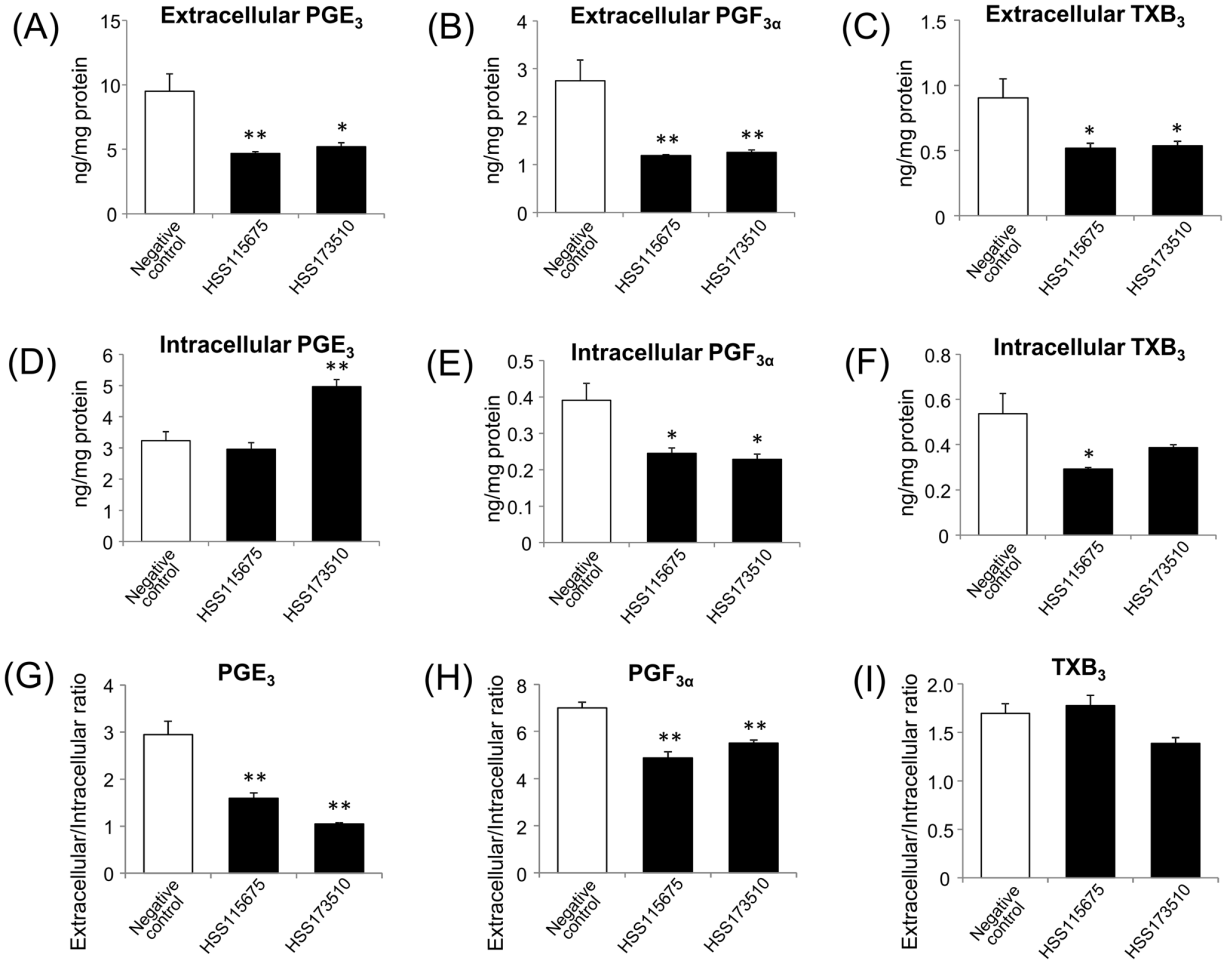
Effects of ABCC4 siRNAs (1 nM) on the transport of 3-series prostanoids in A549 cells. ABCC4 siRNAs (HSS115675 and HSS173510) and negative control siRNA were transfected in A549 cells for 72 h. The A549 cells were then treated with 100 µM EPA for 24 h followed by 10 µM A23187 for 5 min. The extracellular and intracellular levels of (A, D) PGE_3_, (B, E) PGF_3α_, and (C, F) TXB_3_ were measured and (G–H) the ratio of extracellular to intracellular levels were calculated. Each column represents the mean with S.E. (n = 3). Representative experiments are shown. *; *p*<0.05, **; *p*<0.01.

ABCC4 was knocked down by transfection of ABCC4 siRNAs to further evaluate the contribution of ABCC4. As shown in Figure 4, the protein levels of ABCC4 were remarkably decreased at 72 h after transfection. Under this condition, the extracellular levels of the 3-series prostanoids were significantly decreased by approximately 40–50% (Figure 5(A)–(C)). The intracellular levels of PGE_3_ were not changed (HSS115675) or significantly increased to 150% of the negative control (HSS173510) (Figure 5(D)), on the other hand those of PGF_3α_, and TXB_3_ decreased (Figure 5(E)–(F)). The extracellular/intracellular ratio of PGE_3_ and PGF_3α_ was significantly decreased by 50–60% and 20–30%, respectively (Figure 5(G)–(H)). On the other hand, the extracellular/intracellular ratio of TXB_3_ was not significantly decreased (Figure 5(I)).

## Discussion

3-Series prostanoids are mainly synthesized by COX-2, which is highly induced in inflammatory and tumor tissues [12], [31], [32]. In these tissues COX-2 synthesizes proinflammatory and proliferation mediators such as PGE_2_, which promotes transition to chronic inflammation and tumor growth [27]–[29]. To compete with these mediators, the 3-series prostanoids need to be released to the extracellular space rapidly. The permeability of plasma membrane for prostanoids is low because of their carboxylic acid moiety, thus we expected an efflux transporter to be required for rapid 3-series prostanoids release [30]. However, this release mechanism has not been investigated before. The purpose of our study was to elucidate the contribution of ABCC4 to the release of the 3-series prostanoids (PGE_3_, PGF_3α_, and TXB_3_). To investigate whether 3-series prostanoids are substrates of ABCC4, we first carried out vesicular transport studies by using inside-out membrane vesicles prepared from ABCC4-overexpressing HEK293 (HEK293/4.63) cells. We confirmed that our vesicles gave results for 2-series prostanoids (0.9 µM for PGE_2_, 5.3 µM for PGF_2α_, and 8.0 µM for TXB_2_, Table 1) similar to those of previous reports [17], [18]. ATP- and ABCC4-dependent transport of 3-series prostanoids was observed with our vesicles (Figs. 1 and 2) and the transport was reduced by typical substrates and inhibitors of ABCC4 (Table 2), showing that ABCC4 can transport 3-series prostanoids. The *K_m_* values of 3-series prostanoids were higher than those of 2-series prostanoids (Table 1), consistent with the results for their receptors [12]. The difference between 3-series prostanoids and 2-series prostanoids is the presence of a C-17/C-18 double bond, which might make the 3-series prostanoids inflexible and result in a strained conformation in the binding site of ABCC4 [31], [32]. The rank order of the *K_m_* values of the 3-series prostanoids was similar to that of 2-series prostanoids [17], [18]. This difference of the affinity and transport efficiency (*V_max_*/*K_m_*) among the 3-series prostanoids (Table 1) might result from the variation of the chemical properties of their cyclopentane or tetrahydropyrane ring. Further investigations such as the transport by ABCC4 mutants, or the effects of chemical modification of the amino acid residues of ABCC4 showed give more detailed information [33], [34].

Our quantification results showed that ABCC4 inhibitors and ABCC4 knockdown might affect the production of the 3-series prostanoids in A549 cells (Figs. 3 and 5). The total amount of TXB_3_ was significantly decreased in the presence of MK571 (0.79 and 0.31, ng/mg protein for control and MK571, respectively; *p*<0.05 by Dunnett's test), and that of PGF_3α_ was also significantly decreased in the presence of dipyridamole and MK571 (1.01, 0.66, and 0.72 ng/mg protein for control, dipyridamole, and MK571, respectively; *p*<0.05 by Dunnett's test) (Figure 3). In a previous study, we confirmed that dipyridamole, MK571, and probenecid did not change the activity of COX-2 [20]. MK571, also known as a selective CysLT1 receptor inhibitor, reduced the production of PGD_2_ in human mast cell in the presence of leukotriene (LT) D4 and LTE4 [35]. However, 5 min incubation with MK571 was not considered to be not long enough because this effect was caused by alteration of COX-2 expression via ERK pathway. It seems that these inhibitors are likely to affect the production of 3-series prostanoids by unknown ways. In addition, we observed a substantial reduction of the amounts of the 3-series prostanoids after ABCC4 knockdown (Figure 5(A)–(F)). It was reported that the COX-2 gene has a cAMP response element (CRE) in the promoter region, and that COX-2 expression was attenuated in ABCC4 knockout mice because of the reduction of cAMP efflux [19]. However, when we confirmed the expression level of COX-2 protein, COX-2 expression was increased by using HSS173510, one of ABCC4 siRNAs, while using another ABCC4 siRNA (HSS115675) showed reduction of COX-2 protein expression (Figure S1). The reason why this discrepancy between increasing the expression levels of COX-2 protein and decreasing 3-series prostanoid production was caused has been unknown. Therefore, to correct for the difference in the production of 3-series prostanoids, we calculated the extracellular/intracellular ratio and evaluated the contribution of ABCC4 to the release of 3-series prostanoids [19]. The reduction of the extracellular/intracellular ratio of PGE_3_ and PGF_3α_ suggested that ABCC4 might contribute to the release of PGE_3_ and PGF_3α_ (Figure 3(G)–(H), Figure 5(G)–(H)), and that the extracellular/intracellular ratio of PGE_3_ might be decreased more than that of PGF_3α_ due to the difference of their affinity for ABCC4. However, the extracellular levels of PGE_3_ and PGF_3α_ did not show a substantial decrease, despite the use of 50 µM dipyridamole and 50 µM MK571 (Figure 3(A)–(C)), which inhibit strongly the transport of 3-series prostanoids by ABCC4 (Table 2), or marked ABCC4 knockdown (Figure 4, Figure 5(A)–(C)). These results suggest that other transporters, not inhibited by 50 µM dipyridamole and 50 µM MK571, might be involved in the release of PGE_3_ and PGF_3α_. On the other hand, ABCC4 knockdown and MK571 did not decrease the extracellular/intracellular ratio of TXB_3_ (Figure 3(I), Figure 5(I)), suggesting that ABCC4 might not contribute to the release of TXB_3_. Dipyridamole and probenecid produced a reduction of the extracellular/intracellular ratio of TXB_3_ (Figure 3(I)), indicating that transporters, which contribute to the release of TXB_3_, might be inhibited by these inhibitors. It was reported that dipyridamole and probenecid were inhibitors of organic anion transporting polypeptides (OATPs) and organic anion transporters (OATs), respectively [36], [37]. These transporter families were reported to transport PGE_2_ and PGF_2α_ [38], [39]. Previous reports showed the expression of several OATPs and OAT4 mRNA in A549 cells [40], [41], thus these transporters might contribute to the release of TXB_3_. The precise mechanisms involved in TXB_3_ transport remain to be clarified.

In conclusion, we show that PGE_3_, PGF_3α_, and TXB_3_ are new endogenous substrates of ABCC4. Furthermore, inhibition study and RNA interfering study showed the partial contribution of ABCC4 to the release of PGE_3_ and PGF_3α_.

## Supporting Information

Figure S1 **COX-2 protein expression and gross total amounts of 3-series prostanoids in A549 cells.** (A) ABCC4 siRNAs (HSS115675 and HSS173510) and negative control siRNA were transfected in A549 cells for 72 h. Protein (5 µg/lane) was subjected to SDS-PAGE and then transferred onto nitrocellulose membranes. COX-2 and actin were detected with monoclonal COX-2 or actin antibody. (B) Gross total amounts of PGE_3_, PGF_3α_, and TXB_3_ were obtained by addition of intracellular and extracellular amounts of 3-series prostanoids which were acquired from the results shown in Figure 5 in the revised manuscript. Each column represents the mean with S.E. (n = 3).
(TIF)
